# Cardiovascular risk profiles in patients with primary sclerosing cholangitis

**DOI:** 10.1097/MEG.0000000000003146

**Published:** 2026-02-23

**Authors:** J.A.M. Sleutjes, N. van de Pol, A.J. van der Meer, R.A. de Man, C.J. van der Woude, J.E. Roeters van Lennep, A.C. de Vries

**Affiliations:** a Department of Gastroenterology and Hepatology; b Department of Internal Medicine, Erasmus MC Cardiovascular Institute, Erasmus University Medical Center, Rotterdam; c Department of Healthcare Related Education, Radboud University Medical Center, Nijmegen, The Netherlands

**Keywords:** cardiovascular risk factors, ideal cardiovascular health, inflammatory bowel disease, prevention, primary sclerosing cholangitis, screening

## Abstract

**Introduction:**

Chronic inflammation as seen in patients with primary sclerosing cholangitis (PSC) is linked to accelerated development and progression of atherosclerosis. This study aimed to assess atherosclerotic cardiovascular disease (ASCVD) risk and cardiovascular health (CVH) profiles in patients with PSC.

**Methods:**

This cross-sectional study included PSC patients with and without concomitant inflammatory bowel disease (IBD), assessing cardiovascular risk with anthropometric measurements, serum samples, self-reported questionnaires, and medical chart review. The ideal CVH score was calculated according to the American Heart Association guidelines. Comparisons across groups were analyzed using χ^2^ and Mann–Whitney U-test.

**Results:**

Ninety-eight patients with PSC were included [72% male; median age 49 years, interquartile range: 34–61 years], with 65% having concomitant IBD. PSC–IBD patients exhibited higher prevalence of obesity (15% vs 0%; *P*= 0.016), hypertension (35% vs 21%; *P*= 0.069), diabetes (9% vs 3%; *P*= 0.525), active smokers (10% vs 3%; *P*= 0.253) and metabolic syndrome (8% vs 3%; *P*= 0.363), and lower prevalence of hypercholesterolemia (30% vs 50%; *P*= 0.020). Nonideal CVH was more common among PSC–IBD (46% vs 22%; *P* = 0.045). Ten patients had a history of 16 ASCVD events. At time of these events, at 13/16 events patients had one or more traditional ASCVD risk factors of which 77% were modifiable.

**Conclusion:**

One-third of PSC patients exhibit nonideal CVH, particularly PSC-IBD patients. In this cohort, 10% had prior ASCVD with mostly modifiable risk factors at time of the event. These findings suggest that PSC patients might benefit from proactive ASCVD surveillance and risk management.

Primary sclerosing cholangitis (PSC) is a chronic liver disease characterized by inflammation and scarring of the bile ducts leading to cholestasis and ultimately cirrhosis. Prognostic studies in PSC have mainly focused on liver-related complications, liver transplantation, and mortality. However, extrahepatic morbidity and mortality risks, particularly related to atherosclerotic cardiovascular disease (ASCVD), are largely unexplored. Chronic inflammation as seen in PSC patients has been linked to accelerated development and progression of atherosclerosis, similar to observations made in rheumatoid arthritis, SLE, and inflammatory bowel diseases (IBD) [1]. Therefore, this study aimed to assess ASCVD risk and cardiovascular health (CVH) profiles in patients with PSC.

In this cross-sectional single-center study, consecutive PSC patients with and without concomitant IBD were included after informed consent, excluding those with a history of liver transplantation, pregnancy, or lactation. Cardiovascular risk assessment encompassed anthropometrics (blood pressure (BP), body mass index (BMI), and waist and hip circumference), serum sampling (glucose and lipid profiles, including total cholesterol, high-density lipoprotein cholesterol, low-density lipoprotein cholesterol, triglycerides, apolipoprotein-B, and lipoprotein(a)), self-reported questionnaires (alcohol use, smoking, physical activity, dietary habits, and IBD clinical disease activity scores), and medical chart review (a history of ASCVD events, traditional risk factors, and medication use). Metabolic syndrome was defined according to the Adult Treatment Panel III criteria [2], including waist circumference (over 40 inches for men and 35 inches for women), BP (over 130/85 mmHg), triglyceride levels (≥1.7 mmol/l), high-density lipoprotein cholesterol levels (<1.03 mmol/l for men; <1.29 mmol/l for women), and glucose (nonfasting: ≥11.1 mmol/l). Medications were classified into lipid lowering, antihypertensive, glucose regulating, anticoagulative, oral contraceptive, anticholestatic, and IBD immunomodulating agents. Serum biomarkers measured included C-reactive protein, liver enzymes, and platelet count. PSC characteristics were recorded, including disease duration (in years), degree of steatosis (ranging from none to severe: S0–S3), and fibrosis stage (no or mild to advanced fibrosis/cirrhosis: F1–F4) using tissue elastography (FibroScan, Echosens, Paris, France). IBD disease characteristics included disease duration (years), level of fecal calprotectin, colonoscopy findings (within ±1 year), and the Montreal classification specified for Crohn’s disease and ulcerative colitis (age at diagnosis, disease location/extent, and complications such as stenosis and penetration). Clinical IBD activity was assessed using the Harvey Bradshaw Index for Crohn’s disease and the Simple Clinical Colitis Activity Index for ulcerative colitis, with remission defined as a Harvey Bradshaw Index score of less than 5 and active disease as greater than or equal to 5. The ideal CVH score was calculated according to the 2010 American Heart Association concept [3], based on the following metrics: smoking status, BMI, physical activity, diet, BP, total cholesterol, and glucose levels. A scoring system was used, where 2, 1, or 0 points were assigned for ideal, intermediate, or poor outcomes, respectively. The total CVH score ranged from 0 to 14, with scores below 10 or a history of ASCVD indicating nonideal CVH. Comparisons across groups were analyzed using χ^2^ and Mann–Whitney U-test.

A total of 98 patients with PSC [72% males; median age: 49 years; interquartile range (IQR): 34–61 years] underwent cardiovascular risk assessment (Table 1). Sixty-four patients (65%) were diagnosed with concomitant IBD, with 53 (82%) in clinical remission. Median PSC disease duration was 9 years (IQR: 5–15 years). FibroScan performed on 85 (87%) patients diagnosed none/mild, moderate, severe, and advanced fibrosis/cirrhosis in 34, 22, 11, and 33 of patients. Additionally, none, mild, moderate, and severe steatosis was present in 70, 12, 8, and 10%.

**Table 1.** Prevalence of cardiovascular risk factors and events in PSC population

	Total (*n* = 98)	PSC (*n* = 33)	PSC–IBD (*n* = 65)	*P* value
Men	71 (72.4)	21 (61.8)	50 (78.1)	0.062
Median age, years (IQR)	49 (34–61)	52 (38–62)	48 (33–58)	0.151
History of ASCVD^a^	10 (10.2)	2 (5.9)	8 (12.5)	0.334
Traditional risk factors				
Active smokers	7 (7.1)	1 (2.9)	6 (9.4)	0.253
Overweight^b^	28 (28.6)	10 (30.3)	18 (27.7)	0.273
Obesity^c^	10 (10.2)	0 (0)	10 (15.4)	0.016*
Hypertension^d^	29 (29.6)	7 (20.6)	22 (34.4)	0.069
Hypercholesterolemia^e^	36 (36.7)	16 (50.0)	20 (30.3)	0.020*
Diabetes^f^	7 (7.1)	1 (2.9)	6 (9.4)	0.525
Cardiovascular drug use				
Lipid lowering	8 (8.2)	5 (14.7)	3 (4.7)	0.021*
Antihypertensive	21 (21.4)	7 (20.6)	14 (21.9)	0.538
For hypertension	6 (6.1)	-	6 (9.4)	0.072
Anti diabetics	6 (6.1)	-	6 (9.4)	0.094
Anticoagulants	9 (9.2)	3 (8.8)	6 (9.4)	0.742
Anthropometric measurements, median (IQR)				
SBP, mmHg	123 (116–135)	121 (105–126)	125 (121–137)	0.008*
DBP, mmHg	79 (71–84)	73 (66–79)	81 (72–89)	0.002*
BMI, kg/m^2^	24.1 (22.1–27.0)	23.7 (22.1–26.4)	24.5 (22.1–27.9)	0.210
Waist circumference, cm	92 (85–103)	92 (86–101)	93 (85–104)	0.493
WHR	0.91 (0.85–0.95)	0.91 (0.89–0.96)	0.90 (0.85–0.95)	0.528
Median serum levels (*n* = 86) (IQR)				
Glucose, mmol/l	5.4 (5.0–6.0)	5.4 (5.1–6.0)	5.3 (5.0–6.2)	0.747
Total cholesterol, mmol/l	4.9 (3.8–5.6)	5.2 (4.6–5.7)	4.4 (3.6–5.4)	0.008*
HDL-c, mmol/l	1.5 (1.2–2.1)	1.8 (1.3–2.5)	1.4 (1.1–1.9)	0.030*
LDL-c, mmol/l	2.8 (2.1–3.5)	2.8 (2.4–2.6)	2.8 (1.9–3.5)	0.313
Triglycerides, mmol/l	1.1 (0.8–1.5)	1.0 (0.8–1.3)	1.1 (0.9–1.5)	0.112
Apolipoprotein-B, g/l	0.8 (0.6–1.0)	0.8 (0.6–1.0)	0.8 (0.6–0.9)	0.884
Lp(a), nmol/l	11 (7–49)	11 (7–55)	12 (7–30)	0.669
Non-HDL-c	3.1 (2.3–3.8)	3.3 (2.6–4.2)	2.9 (2.2–3.7)	0.173
Remnant cholesterol	0.2 (0.1–0.5)	0.2 (0.1–0.5)	0.2 (0.1–0.5)	0.661
Metabolic syndrome^f^	6 (6.1)	1 (2.9)	5 (7.8)	0.363

Frequencies are described as numbers (percentages) unless stated otherwise. ASCVD, atherosclerotic cardiovascular disease; BMI, body mass index; DBP, diastolic blood pressure; HDL-c, high-density lipoprotein cholesterol; IBD, inflammatory bowel disease; IQR, interquartile range; LDL-c, high-density lipoprotein cholesterol; PSC, primary sclerosing cholangitis; SBP, systolic blood pressure; WHR, waist-to-hip-ratio.

a Angina pectoris, myocardial infarction, percutaneous transluminal coronary angioplasty/coronary artery bypass grafting, transient ischemic attack, cerebrovascular accident, or heart failure.

b Overweight is defined as BMI = 25–29.9 kg/m^2^ and obesity as BMI > 30 kg/m^2^.

c SBP > 140 mmHg or DBP > 90 mmHg, or use of antihypertensive drugs indicated for diagnosis of hypertension.

d Total cholesterol >5 mmol/l, or indication of lipid lowering drugs for hypercholesterolemia.

e Nonfasting glucose ≥ 11.11 mmol/l or use of antidiabetic drugs for diagnosis of diabetes type I or II.

f Minimum of 3 out of 5 of the following criteria: (a) nonfasting serum glucose ≥ 11.1 mmol/l or diabetes drugs, (b) SBP ≥ 130 mmHg or DBP ≥ 85 mmHg or antihypertensive drug use indicated for hypertension, (c) HDL-c ≤ 1.04 mmol/l for men or ≤1.29 mmol/l for women, (d) serum triglycerides ≥ 1.7 mmol/l, and (e) waist circumference > 102 cm for men and >88 cm for women.

The prevalence of current smokers was 2%, overweight 29%, obesity 10%, hypertension 30%, hypercholesterolemia 37%, diabetes 7%, and 14% had a positive family history of ASCVD. Six (6%) patients fulfilled the criteria of metabolic syndrome. Nonideal CVH was observed in 37% of patients, with most prevalent ideal CVH metrics being nonsmoking (93%) and normoglycemia (91%), and most prevalent nonideal CVH metrics, including diet (81%) and BP (79%).

PSC–IBD patients compared with PSC patients exhibited a higher prevalence of obesity (15% vs. 0%; *P* = 0.016), hypertension (35% vs. 21%; *P* = 0.069), diabetes (9% vs. 3%; *P* = 0.525), active smokers (10% vs. 3%; *P* = 0.253), and metabolic syndrome (8% vs. 3%; *P* = 0.363), and a lower prevalence of hypercholesterolemia (30% vs. 50%; *P*= 0.020). Nonideal CVH was more common among PSC–IBD patients as compared to PSC patients (46% vs. 22%; *P* = 0.045)(Fig. 1).

**Fig. 1.**
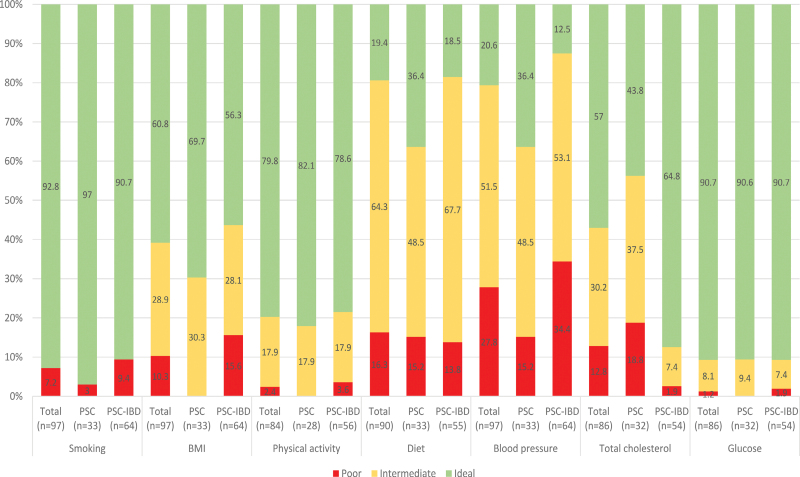
Prevalence of CVH status and metrics for total, PSC and PSC–IBD populations. Handled definitions per metric: smoking, poor: current, ideal: never/former; BMI (kg/m^2^), poor: ≥30, intermediate: 25–<30, ideal: <25; BP (mmHg), poor: systolic ≥140 or diastolic ≥90, intermediate: systolic 120–139 or diastolic 80–89 mmHg, or treated to goal, ideal: systolic <120 and diastolic <80; physical activity, poor: inactive, intermediate moderately active, ideal: active; diet (number of ideal components), poor: 0–1, intermediate: 2–3, ideal: 4–5; total cholesterol level (mmol/l), poor: ≥6.2, intermediate: 5.2–6.2, ideal: <5.2; and glucose (mmol/l, nonfasting), poor: ≥11.1, intermediate: 7.8–11.11, ideal: <7.8. The sum of the metrics yields an overall score between 0 and 14, with nonideal being defined as CVH score <10 or a history of an ASCVD event. ASCVD, atherosclerotic cardiovascular disease; BP, blood pressure; CVH, cardiovascular health; IBD, inflammatory bowel disease; PSC, primary sclerosing cholangitis.

Ten (10%) patients had a history of, in total, 16 ASCVD events, with the first event occurring at a median age of 53 years (IQR: 49–57 years), more frequently in PSC–IBD as compared to PSC patients (13% vs. 6%, *P*= 0.335). Notably, at time of these events, 7/10 patients had extensive colonic disease involvement (L2/L3: colonic/ileoconic disease or E3: pancolitis) and 7/10 advanced fibrosis/cirrhosis. Moreover, at 13/16 events patients had greater than or equal to 1 traditional ASCVD risk factor of which 77% were modifiable, that is, smoking (8%), overweight (31%), obesity (23%), hypertension (23%), and hypercholesterolemia (23%) (Table 2).

**Table 2.** Description of patient and disease characteristics at time of the ASCVD event

Patient number	ASCVD type	Year	Age, years	Sex	Diagnosis	Disease duration, y		Montreal classification	Therapy	FibroScan	CAP	BMI (kg/m^2^)	Traditional risk factors
						PSC	IBD						
1	TIA	2006	52	Female	PSC–IBD	0.5	27	E3	5-ASA	F1	-	27.8	Overweight; FH
	MI, PTCA, HF	2015	61			9	36		5-ASA, bud, UDCA, anti-coag, B-blocker, statin	F2		26.2	Overweight; hypertension; DM II (2015); FH
2	TIA	2016	38	Male	PSC–IBD	15	15	A2L2B2/E3	5-ASA, UDCA	F4	<5%	23.6	DM II
3	HF	2012	63	Female	PSC–IBD	6	6	A3L3B1	5-ASA, UDCA	F1	34-66%	27.9	FH; overweight; post-menopausal
4	HF	2008	58	Male	PSC	>	–	–	–	–	–	23.8	FH
5	MI, PTCA	2013	44	Male	PSC–IBD	15	29	A1L3B1	5-ASA, UDCA	F4	<5%	22.1	FH
	NSTEMI	2018	49			20	34	?	5-ASA, UDCA, statin, anti-coag	?	?	21.9	FH; hypercholesterolemia
6	MI, PTCA	2013	57	Male	PSC–IBD	17	17	E3	5-ASA, UDCA	?	<5%	24.5	Hypercholesterolemia
	PTCA	2018	62			22	22		5-ASA, UDCA, anti-coag, statin	F3-4	<5%	24.5	–
	PTCA	2019	63			23	23		5-ASA, UDCA, anti-coag, statin	F3-4	<5%	24.8	–
7	HF	2020	64	Male	PSC–IBD	5	41	A2L3B?	UDCA	F1	34-66%	22.7	FH
8	MI, CABG	2007	49	Male	PSC	>	–	–	**–**	**–**	**–**	26.0	FH; Active smoker; overweight
9	HF	2009	54	Female	PSC–IBD	13	15	E2	Glucose regulating	F0-1	>66%	33.9	Obesity; DM II; pregnancy hypertension; PAPVR
	PTCA	2011	56			15	17		Glucose regulating, diuretic	F1	>66%	33.7	Obesity; DM II; pregnancy; hypertension; hypertension
	TIA (multiple)	2017–2021	62			21	23		Glucose regulating, diuretic, B-blocker	F1	>66%	37.7	Hypercholesterolemia; morbid obesity; OSAS
10	HF	2019	50	Female	PSC–IBD	?	22	E3	Diuretic, pred, vedo	F4	>66%	19.6	–

The Montreal classification was used to define disease extent, in Crohn’s disease by A standing for age at diagnosis (A1: <17 years, A2 : 17-39 years, A3 ≥ 40 years), L for disease location (L1: ileal, L2: colonic, L3: ileocolonic, L4: upper disease), and B for behavior (B1: nonstricturing and nonpenetrating, B2: stricturing, B3: penetrating), and in ulcerative colitis by E for extent (E1: proctitis, E2: left-sided colitis, E3: pancolitis).

5-ASA, 5-aminosalicylic acids; anti-coag, anticoagulant; bud, budesonide; CABG, coronary artery bypass grafting; DM II, diabetes mellitus type 2; FH, positive familial history; HF, heart failure; IBD, inflammatory bowel disease; MI, myocardial infarction; NSTEMI, non-ST elevation myocardial infarction; OSAS, obstructive sleep apnea syndrome; PAPVR, partial anomalous pulmonary venous return; PTCA, percutaneous transluminal coronary angioplasty; PSC, primary sclerosing cholangitis; pred, prednisone; UDCA, ursodeoxycholic acid; vedo, vedolizumab; TIA, transient ischemic attack; >, PSC/IBD diagnosis after the ASCVD event; ?, unknown.

This study demonstrates that overweight, hypertension, and hypercholesterolemia affect nearly one-third of patients with PSC, and 37% had nonideal CVH. Additionally, the majority of patients had modifiable traditional risk factors at the time of their ASCVD event, suggesting targeted cardiovascular screening could improve CVD outcomes in this population.

Data regarding cardiovascular risk related to PSC are limited and inconsistent. One prospective study comparing 678 PSC patients to age/sex-matched controls found a neutral risk for ischemic heart disease (relative risk: 0.90, 95% CI: 0.55–1.48) and only slightly elevated for cerebrovascular disease (relative risk: 1.74, 95% CI: 1.08–2.78) [4]. A meta-analysis on ASCVD risk in a mixed population of liver disease showed that pretransplant obesity [odds ratio (OR): 2.44], hypertension (OR: 1.96), and particularly diabetes (OR: 4.03) were related to new-onset metabolic syndrome following liver transplantation [5]. These data, together with our observation that 10% of pretransplant patients had ASCVD events and 37% nonideal CVH, highlight the importance of earlier CVD screening in pretransplant PSC patients.

The prevalence of modifiable traditional risk factors in our population differs from general population data, with higher prevalence of diabetes (7% vs. 2.6%), hypertension (30% vs. 22.5%), hypercholesterolemia (37% vs 12.8%) alongside lower prevalence of smoking (2% vs. 21.5%) and overweight (39% vs. 54%) [6]. An interesting finding was the high percentage of patients with extensive colitis at time of ASCVD event. Although limited by low numbers, this aligns with prior observations in IBD [1]. This might be explained by systemic inflammation exacerbating atherosclerosis, a procoagulative state, and the role of the microbiome in ASCVD development.

Current PSC guidelines (European Association for the Study of the Liver/American Association for the Study of Liver Diseases) do not recommend routine cardiovascular screening, focusing assessment primarily on patients with end-stage liver disease considered for or after liver transplantation [7,8]. Consequently, proper identification of PSC patients at increased CVD risk remains inadequate. Despite limited power and lack of multivariable analysis that restrict firm conclusions, addressing modifiable risk factors within the PSC population may still be beneficial. The CVH serves as an accessible, easy-to-use screening tool that can guide risk factors identification and intervention. Effective prevention may involve lifestyle modification, including dietary changes and physical exercise.

For managing hypercholesterolemia, statins and ezetimibe appear safe with close monitoring of liver biochemistry [9]. Long-term use of ursodeoxycholic acid significantly reduces total cholesterol and low-density lipoprotein levels compared to placebo [10]. It is important to recognize the ongoing debate regarding clear correlations between cholesterol levels and ASCVD risk in cholestatic liver disease, arising from factors such as impaired cholesterol metabolism; the pandemic of metabolic syndrome affecting PSC population; and the varied clinical presentation influenced by comorbidities such as IBD. Consequently, the decision to treat hypercholesterolemia remains an individual choice.

In conclusion, this study showed that one-third of PSC patients exhibit nonideal CVH, even higher among PSC patients with IBD. In this cohort, 10% had prior ASCVD with mostly modifiable risk factors at time of the event. These findings suggest that PSC patients might benefit from proactive ASCVD surveillance and risk management. Further studies should focus on the impact of early CVH assessment and intervention on both short- and long-term ASCVD outcomes.

## Acknowledgements

We are indebted to the staff and participants of the CARE-PSC Study for their important contributions.

This study was approved by the Medical Ethics Committee (MEC-2019-0408) and conducted in compliance with the Declaration of Helsinki. Written informed consent was obtained from all participants.

The individual patient data underlying this article will be shared on reasonable request to the corresponding author.

### Conflicts of interest

A.J.v.d.M. received honoraria from Gilead, CymaBay, Ipsen, and Mirum, fees for promotional material from Dr. Falk, and research funding from Gilead, CymaBay, Intercept, Mirum, and Ipsen. A.C.d.V. received unrestricted research grants from Takeda, Lilly, Janssen, and Pfizer, outside the submitted work. For the remaining authors, there are no conflicts of interest.
